# Effectiveness of Boswellia and Boswellia extract for osteoarthritis patients: a systematic review and meta-analysis

**DOI:** 10.1186/s12906-020-02985-6

**Published:** 2020-07-17

**Authors:** Ganpeng Yu, Wang Xiang, Tianqing Zhang, Liuting Zeng, Kailin Yang, Jun Li

**Affiliations:** 1 The Department of Orthopaedics, People’s Hospital of Ningxiang City, Ningxiang, 410600 Hunan Province China; 2 Graduate College, Guilin Medical University, Guilin, Guangxi Province China; 3 Department of Rheumatology, Affiliated Hospital of Guilin Medical University, Guilin, Guangxi Province China; 4 Graduate College, University of South China, Hengyang, Hunan Province China; 5 Department of Cardiology, The First Affiliated Hospital of University of South China, Hengyang, Hunan Province China; 6 Graduate College, Chinese Academy of Medical Sciences & Peking Union Medical College, Beijing, China; 7 Hunan University of Chinese Medicine, Changsha, 410208 Hunan Province China

**Keywords:** Boswellia, Boswellia extract, Osteoarthritis, Systematic review, Meta-analysis

## Abstract

**Background:**

Osteoarthritis (OA) is the commonest form of inflammatory joint disease. Unfortunately, to date, there is no appropriate treatment for OA. *Boswellia serrata* was considered as a potent anti-inflammatory, anti-arthritic and analgesic agent that may be a drug for OA.

**Methods:**

In this meta-analysis, data from randomized controlled trials were obtained to assess the effects of Boswellia or its extract versus placebo or western medicine in patients with OA. The primary outcomes included visual analogue score (VAS), WOMAC pain, WOMAC stiffness, WOMAC function and lequesne index.

**Result:**

Seven trials involving 545 patients were included. Compared with the control group, Boswellia and its extract may relieve the pain [VAS: (WMD -8.33; 95% CI -11.19, − 5.46; *P*<0.00001); WOMAC pain: (WMD -14.22; 95% CI -22.34, − 6.09; P = 0. 0006)] and stiffness [WOMAC stiffness: (WMD -10.04; 95% CI -15.86, − 4.22; P = 0. 0007)], and improve the joint’s function [WOMAC function: (WMD -10.75; 95% CI -15.06, − 6.43; *P*<0. 00001); lequesne index: (WMD -2.27; 95% CI -3.08, − 1.45; *P*<0. 00001)].

**Conclusion:**

Based on current evidence, Boswellia and its extract may be an effective and safe treatment option for patient with OA, and the recommended duration of treatment with Boswellia and its extract is at least 4 weeks.

## Background

Osteoarthritis (OA) is an inflammatory joint disease that mainly damages articular cartilage. It is characterized by the degradation of articular cartilage and involving the entire joint tissue, which eventually leads to joint degeneration, fibrosis, fracture, defect and damage to the entire articular surface [1–3]. Epidemiological studies show that there are currently 355 million people with arthritis worldwide, and arthritis has become the world’s number one disabling disease. In China, as of 2015, there were 120 million people with arthritis in mainland China, with an incidence rate of about 13%, of which the number of people suffering from OA is the largest [4]. The management of patients with knee OA is mainly based on individualized and gradient treatment for the condition and severity of arthritis [5–7]. Among them, the drugs for OA pain are mainly non-steroidal anti-inflammatory drugs (NSAID) and specific (COX-2) cyclooxygenase II inhibitors [8]. However, these drugs may be expensive or cause stomach bleeding, and cannot repair cartilage and treat subchondral damage [8–10]. Therefore, due to the high incidence of NSAID-related adverse events, there is an urgent need for a safer and effective alternative to OA.

Boswellic acid is the active ingredient in *Boswellia serrata*; it has shown significant pharmacological activity in the treatment of inflammatory diseases such as rheumatoid arthritis, chronic bronchitis, asthma and chronic inflammatory bowel diseases (ulcerative colitis and Crohn’s disease) [11, 12]. Current research showed that 3-O-Acetyl-11-keto-beta-boswellic acid (AKBA) is the one boswellic acid with strong pharmacological activity; for example, AKBA has a powerful inhibitory effect on 5-lipoxygenase (5-LOX) [13, 14]. Clinical studies have shown that *Boswellia serrata* extract not only has anti-inflammatory and anti-arthritis properties, but also improves pain and physical function [15–18]; in vitro experiments also show that *Boswellia serrata* extract can inhibit the expression of inflammatory factors such as adhesion molecules [19–23]. With regard to the safety of *Boswellia serrata*, studies showed that *Boswellia serrata* extract (such as 5-Loxin and Aflapin) does not have toxic side effects at higher doses [23–25]. These indicate that the active compound of Boswellia extract (AKBA) is safe based on current evidence [23, 25].

As potential anti-inflammatory drugs for OA, the efficacy of Boswellia and Boswellia Extract have been reported in a lot of clinical trials [14–17, 24, 26]. Since 2003 [15], many trials have explored the feasibility of Boswellia and its extracts for the treatment of OA. In the meantime, the latest randomized controlled trials (RCTs) have assessed the benefits and adverse events of Boswellia and its extract for OA. To the best of our knowledge, this is the first systematic review and meta-analysis estimating the effectiveness and safety of Boswellia and its extracts for OA. Therefore, we hope that the results of this systematic review and meta-analysis will provide better evidence for the clinical application of Boswellia and its extract in the treatment of OA.

## Why it is important to do the review

Boswellia and its extract have theoretical benefits for cultured cells in vitro and experimental animals [19, 21–23, 25, 27]; although several meta-analyses have widely analyzed the effect of herb or dietary supplements on OA [28, 29], to our knowledge, there is no systematic review and meta-analysis focused on assessing the efficacy of Boswellia and its extract on OA. For example, Liu et al. [28] conducted a systematic review about dietary supplements for patients with osteoarthritis; Of the studies it included, only three RCTs were about Boswellia and its extract. Meanwhile, its conclusions about Boswellia and its extract on OA failed to provide valuable reference information for clinical decision making. Another systematic review [29] only analyzed the RCTs before 2013. In 5 years, more RCTs about Boswellia and its extract on OA have been completed, which means that relevant conclusions need to be updated or confirmed. Therefore, this systematic review and meta-analysis focus on and summarizes available evidence from RCTs about the role of Boswellia and its extract in OA.

## Materials and methods

### Protocol

Systematic reviews and meta-analysis are carried out strictly in accordance with the protocol (CRD42018086785) and the PRISMA-guidelines (see Supplementary Materials) [30].

### Search strategy and selection criteria

Web of Science, the Chinese Science and Technology Periodical Database (VIP), Wan Fang Database (Chinese Ministry of Science & Technology), EMBASE, Medline Complete, Chinese Biomedical Database (CBM), ClinicalTrials, the China National Knowledge Infrastructure Databases (CNKI), Pubmed, Cochrane Library (Until Issue 2, 2018) were searched with keywords “boswellic acid”, “Boswellia”, “Shallaki”, “Salai”, “aflapin”, “5-loxin” and “osteoarthritis”. The search period is from the establishment of the journal to January 2018.

### Selection criteria

#### Participants

Human with specified diagnosis criteria of OA.

#### Intervention methods

Boswellia or its extract.

#### Comparison methods

Other treatments for OA, such as placebo or conventional medicine;

#### Outcomes

(1) Primary outcomes: visual analogue score (VAS), WOMAC pain, WOMAC stiffness, WOMAC function and lequesne index. (2) Secondary outcomes: WOMAC pain, WOMAC stiffness, WOMAC function at different points in time.

#### Study design

Randomized controlled trials (RCTs).

### Information retrieval and data extraction method

Three researchers (Ganpeng Yu, Liuting Zeng and Kailin Yang) independently screened the literature based on protocol (CRD42018086785). The discrepancies between the three researchers would be resolved through discussion of all researchers. Literature retrieval is carried out according to the search strategy, the search strategy of Pubmed is shown in Table 1.

**Table 1:** Search Strategy for Pubmed

Database	Search Strategy
Pubmed	(boswellic acid OR Boswellia OR *Boswellia sacra* OR *Boswellia serrata* OR *Boswellia carteri* OR Boswellia carterii OR Shallaki OR Salai OR aflapin OR 5-loxin) AND (Osteoarthritis OR Osteoarthritides OR Osteoarthrosis OR Osteoarthroses OR Arthritis, Degenerative OR Arthritides, Degenerative OR Degenerative Arthritides OR Degenerative Arthritis OR Osteoarthrosis Deformans) AND (randomized controlled trial [pt] OR controlled clinical trial [pt] OR placebo [tiab] OR drug therapy [sh] OR trial [tiab] OR groups [tiab] OR clinical trials as topic [mesh: noexp] OR Clinical Trial OR random* [tiab] OR random allocation [mh] OR single-blind method [mh] OR double-blind method [mh] OR cross-over studies) NOT (animals [mh] NOT humans [mh])

After literature retrieval, three researchers (Ganpeng Yu, Liuting Zeng and Kailin Yang) extracted data from the included literature according to a pre-designed table (including author, year of publication, number of cases, inclusion and exclusion criteria of patients, duration of intervention, etc.). The discrepancies between the three researchers would be resolved through discussion of all researchers. The missing data in the literature would be obtained by contacting the author. If the author cannot be contacted, it would be estimated by the method provided by Cochrane Handbook 5.1.0 [31].

### Literature quality evaluation methods

The Cochrane collaboration’s tool for assessing risk of bias provided by Cochrane Handbook 5.1.0 was used to evaluate the quality of the RCTs [32]. The evaluation content includes random sequence generation, allocation result concealment, blind method, incomplete result data, selective report and other biases. Four researchers (Ganpeng Yu, Wang Xiang, Tianqing Zhang, Liuting Zeng and Kailin Yang) independently assessed the quality of the literature. The discrepancies between the three researchers would be resolved through discussion of all researchers.

### Statistical analysis

The RevMan5.3 software provided by the Cochrane collaboration network was used for system evaluation and meta-analysis. Enumeration data is represented by risk ratio (RR) and 95% confidence interval (CI), and measurement data is represented by mean deviation (MD) and CI. The heterogeneity between the results of the studies was tested by X^2^. If the heterogeneity of the study is low (P > 0.10, I^2^ < 50%), the fixed effect model is used for analysis; otherwise (P < 0.10, I2 > 50%), the source of heterogeneity is analyzed first and then subgroup analysis or random effect model is adopted.

## Results

### Results of the search

**Fig. 1:**
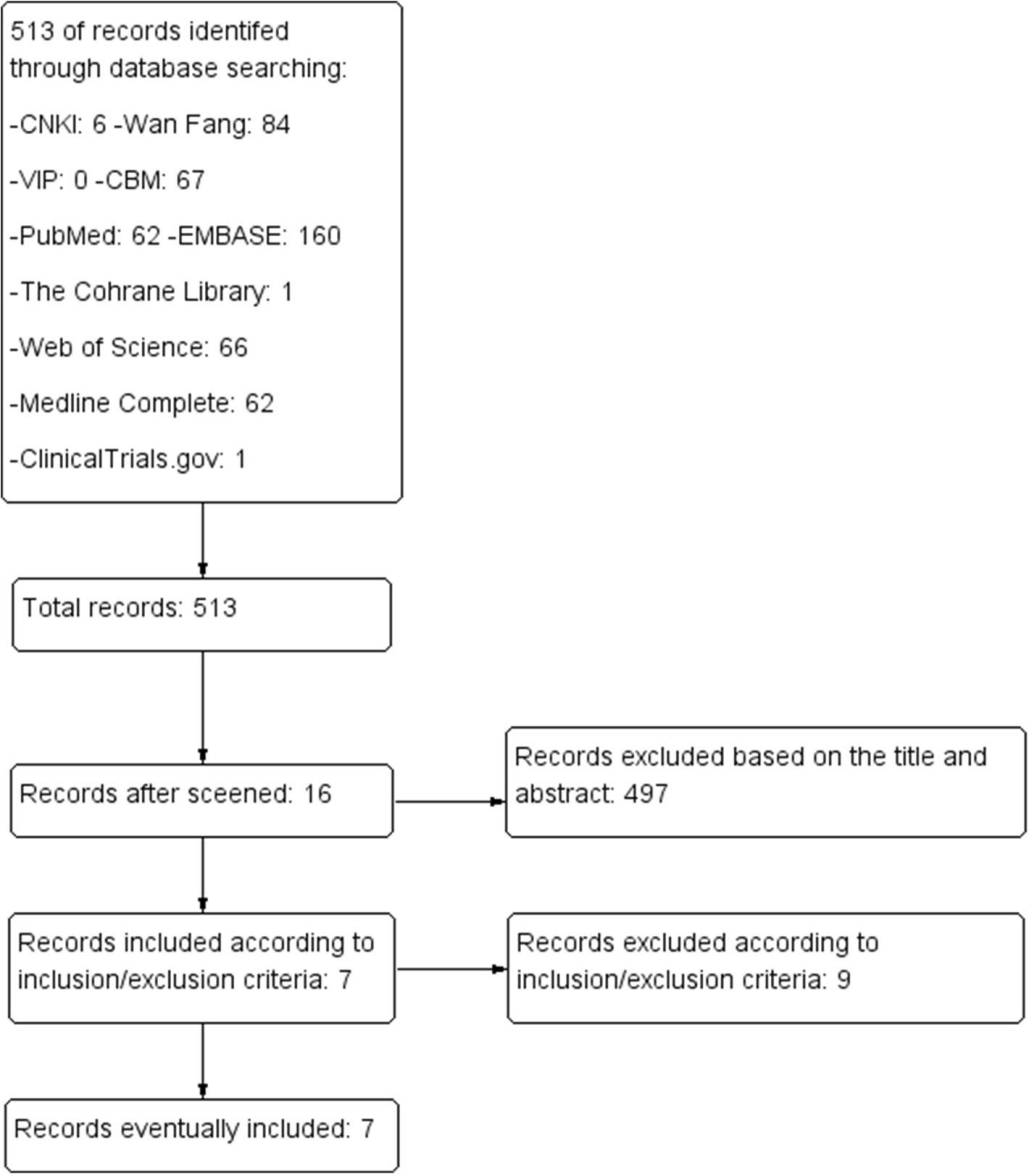
Flow diagram of searching and article selection

The total records identified through database searching and other sources were 513. Four hundred ninety-seven (497) were excluded based on the title and abstract and 16 for more detailed evaluation. After that, 7 studies were included [24, 26, 33–37], while 9 studies were excluded [15–18, 38–42] according to the inclusion criteria (Fig. 1). The 9 studies were Kimmatkar 2003 [15], Chopra 2004 [38], Shah 2010 [17], Chopra 2013 [39], Perera 2014 [16], Belcaro 2015 [40], Bolognesi 2016 [41], Gupta 2011 [18], Badria 2002 [42].

### Description of included trials

A total of 7 RCTs with 545 participants were included. Sengupta’s RCTs consist of 2 trial groups and 1 control group [24, 26]. According to the method of Cochrane Handbook 5.1.0, the control group was divided into two groups, matching the two trial groups (Sengupta 2008a and Sengupta 2008b; Sengupta 2010a and Sengupta 2010b) [31]. The characteristics of the RCTs were shown in Tables 2 and 3.

**Table 2:** The characteristics of the included studies

Study	Country	Sample size		Intervention		Mean age (years)	
		Trial group	Control group	Trial group	Control group	Trial group	Control group
Vishal 2011 [33]	India	30	29	Aflapin 100 mg	Placebo 100 mg	53.2 ± 6.5	55.3 ± 8.8
Sengupta 2008 [26]	India	47	23	5-Loxin 100 mg or 250 mg	Rice bran (placebo)	52.79 ± 8.47	52.43 ± 9.65
Sengupta 2010 [24]	India	38	19	Aflapin 100 mg or 5-Loxin 100 mg	Excipients (placebo) 100 mg	52.4 ± 8.87	52.4 ± 7.5
Haroyan 2018 [34]	Armenia	67	68	Boswellic acid 150 mg + curcuminoids 350 mg	Excipients (placebo) 500 mg	57.91 ± 9.02	56.04 ± 8.55
Karimifar 2017 [35]	Iran	26	26	Boswellia + Elaeagnus Extraction	Ibuprofen	52.0 ± 8.74	52.96 ± 8.57
Notarnicola 2016 [36]	Italty	54	58	Boswellic acid 15 mg + Methylsulfonylmethane 10,000 mg	Glucosamine sulfate 3000 mg	58.7 ± 12.5	59.5 ± 13.7
Notarnicola 2011 [37]	Italty	30	30	Boswellic acid 7.5 mg + Methylsulfonylmethane 5000 mg	Placebo	63.4 ± 8.2	60.2 ± 8.6

**Table 3:** The characteristics of the included studies

Study	Inclusion criteria	Exclusion criteria	Relevant outcomes	Duration
Vishal 2011 [33]	1. Participants must understand risks and benefits of the protocol and able to give informed consent 2. Male and female subjects of 40–80 years of age 3. Females of child bearing potential must agree to use an approved form of birth control and have a negative pregnancy test result 4. Unilateral or bilateral OA of the knee for more than 3 months 5. Visual Analogue Scale (VAS) score during the most painful knee movement between 40 and 70 mm after 7 day withdrawal of usual medication 6. Lequesne’s Functional Index (LFI) score greater than 7 points after 7 days of withdrawal of usual medication 7. Ability to walk 8. Availability for the duration of the entire study period	1. History of underlying inflammatory arthropathy or severe rheumatoid arthritis (RA) 2. Hyperuricemia (greater than 440 umol/L) and/or past history of gout 3. Recent injury in the area affected by OA of the knee (past 4 months) and expectation of surgery in the next 4 months 4. Intra-articular corticosteroid injections within the last 3 months 5. Hypersensitivity to non-steroidal anti-inflammatory drugs (NSAIDs), abnormal liver or kidney function tests, history of peptic ulceration and upper gastrointestinal (GI) haemorrhage, congestive heart failure, hypertension, hyperkalemia 6. Major abnormal findings on complete blood count, history of coagulopathies, haematological or neurological disorders 7. High alcohol intake (greater than 2 standard drinks per day) 8. Pregnant, breastfeeding or planning to become pregnant during the study 9. Use of concomitant prohibited medication other than ibuprofen 10. Obesity: body mass index (BMI) more than 30 kg/m^2	VAS, WOMAC pain subscale, WOMAC stiffness subscale, WOMAC function subscale, Lequesne Index, adverse events	4 weeks
Sengupta 2008 [26]			VAS, WOMAC pain subscale, WOMAC stiffness subscale, WOMAC function subscale, Lequesne Index, MMP-3, adverse events	12 weeks
Sengupta 2010 [24]			VAS, WOMAC pain subscale, WOMAC stiffness subscale, WOMAC function subscale, Lequesne Index, adverse events	12 weeks
Haroyan 2018 [34]	Patients with a diagnosis of degenerative hypertrophic osteoarthritis of the knee (M17, according to International Classification of Diseases (ICD-10) of bone joints, verified by radiography (Grade 1–3 by Kellgren-Lawrence radiographic grades).	1. subjects with inflammatory and any secondary arthritis 2. moderate and severe synovitis (grades 2 and 3) tear of meniscus 3. chronic diseases of the kidneys, liver, gastrointestinal, cardiovascular, endocrine and nervous systems 4. allergic anamnesis and drug intolerance 5. pregnant or nursing 6. history of substance abuse 7. subjects taking non-steroidal anti-inflammatory drugs and analgesics within 2 weeks prior to the study 8. subjects taking glucosamine sulfate, chondroitin sulfate, intra-articular hyaluronate, systemic or intra-articular glucocorticoids within 3 months prior to the study	WOMAC pain subscale, WOMAC stiffness subscale, WOMAC function subscale, adverse events	12 weeks
Karimifar 2017 [35]	1. age of 40 to 80 years 2. knee osteoarthritis in at least one knee for at least 6 months based on ACR (American College of Rheumatology) diagnostic criteria 3. pain score > 4 based on VAS (visual analog scale) 4. LPFI (Lequesne Pain and Function Index) > 7 5. serum CRP (C-reactive protein) < 10 mg/dl and ESR (erythrocyte sedimentation rate) < 20 mg/dL, (6) grade 2 or 3 of KellgrenLawrence scale in knee radiography obtained within the last 6 months 7. free of liver, renal, or cardiac dysfunction 8. no use of intra-articular glucocorticoids or hyaluronic acid preparations within the last 3 months 9. no use of systemic glucocorticoids within the last 3 months 10. free of all other bone and joint disorders including rheumatoid arthritis and gout 11. free of peptic ulcer disease 12. no knee arthroscopic procedure within the last 3 months 13. not being pregnant or lactating (for women).	1. irregular use of capsules (less than 80% of total capsules) 2. no use of capsules for at least 3 days	VAS, Lequesne Index, adverse events	4 weeks
Notarnicola 2016 [36]	1. a diagnosis of OA of the knee according to the criteria of the American College of Rheumatology 2. grade 3 Kellgren and Lawrence radiographic staging,16 in which the severity of the arthritis is assessed on a scale in the range of 0–4, hypothesizing a sequential evolution from the manifestation of osteophytes through a reduction in the width of the joint space, to subchondral sclerosis and finally the formation of cysts 3. frequent joint pain (several days a week) for at least 6 months before recruitment 4. pain in the knee, scored at least 4 cm on a 10 centimetric visual analogic scale (VAS) (from moderate to severe pain), where 0 means no pain and 10 is the worst pain possible 5. a score of > 2 on the Lequesne pain-function index (LI).18 The LI is an OA-specific validated questionnaire that poses a series of questions about pain in the knee (five questions on a scale from 0 to 2, where 0 indicates no pain and 2 intense pain), functional limitation (four questions, using the same scale), and maximum walking distance (one question, with a score from 0 to 6, where 0 indicates the ability to walk for an unlimited distance and 6, the inability to cover 100 m). The maximum worst final score is 24	1. previous surgery of the affected knee 2. disease processes such as rheumatoid arthritis, autoimmune diseases, systemic diseases, and tumors 3. severe obesity (BMI > 40 kg/m2) 4. meniscal or ligament injuries 5. allergy to shellfish 6. altered blood chemistry and kidney, liver, and metabolic (diabetes mellitus) function 7. intra-articular hyaluronic acid/cortisone infiltrations to the affected knee within 3 months before the start of the study 8. systemic cortisone treatment taken within 3 months before the start of the study; 9.supplements (glucosamine, chondroitin sulfate, bromeline, etc.) taken within 3 months before the start of the study (patients were also informed that they were not to be taken for the following 6 months)	VAS, Lequesne Index	24 weeks
Notarnicola 2011 [37]	1. men and women > 45 and < 85 years of age; 2. a diagnosis of OA of the knee according to the criteria of the American College of Rheumatology 3. grade 3 Kellgren and Lawrence radiographic staging,13 in which the severity of the arthritis is assessed on a scale from 0 to 4, hypothesizing a sequential evolution from the manifestation of osteophytes through a reduction in the width of the joint space, to subchondral sclerosis and fnally the formation of cysts; 4. frequent joint pain (several days a week) for at least 6 months before recruitment; 5. pain in the knee, scored at least 2 cm on a 10 centimetric visual analogic scale (VAS), where 0 means no pain and 10 is the worst pain possible; 6. a score of > 2 on the Lequesne pain-function index (LI).14 The LI is a disease-specific validated questionnaire that poses a series of questions about pain in the knee (five questions on a scale from 0 to 2, where 0 indicates no pain and 2 intense pain), functional limitation (four questions, using the same scale) and maximum walking distance (one question, with a score from 0 to 6, where 0 indicates the ability to walk for an unlimited distance and 6, the inability to cover 100 m). The maximim worst fnal score was 24.	1. previous surgery of the affected knee; 2. disease processes such as rheumatoid arthritis, autoimmune diseases, systemic diseases, and tumors; 3. obesity (BMI > 30 kg/m2); 4. altered blood chemistry and kidney, liver, and metabolic (diabetes mellitus) function; 5. intra-articular hyaluronic acid/cortisone infiltrations to the affected knee within 3 months before the start of the study; 6. systemic cortisone treatment taken within 3 months before the start of the study; 7. supplements (glucosamine, chondroitin sulfate, bromeline, etc) taken within 3 months before the start of the study (patients were also informed that they were not to be taken for the following 6 months).	VAS, Lequesne Index, adverse events	24 weeks

### Risk of Bias in included studies

**Fig. 2:**
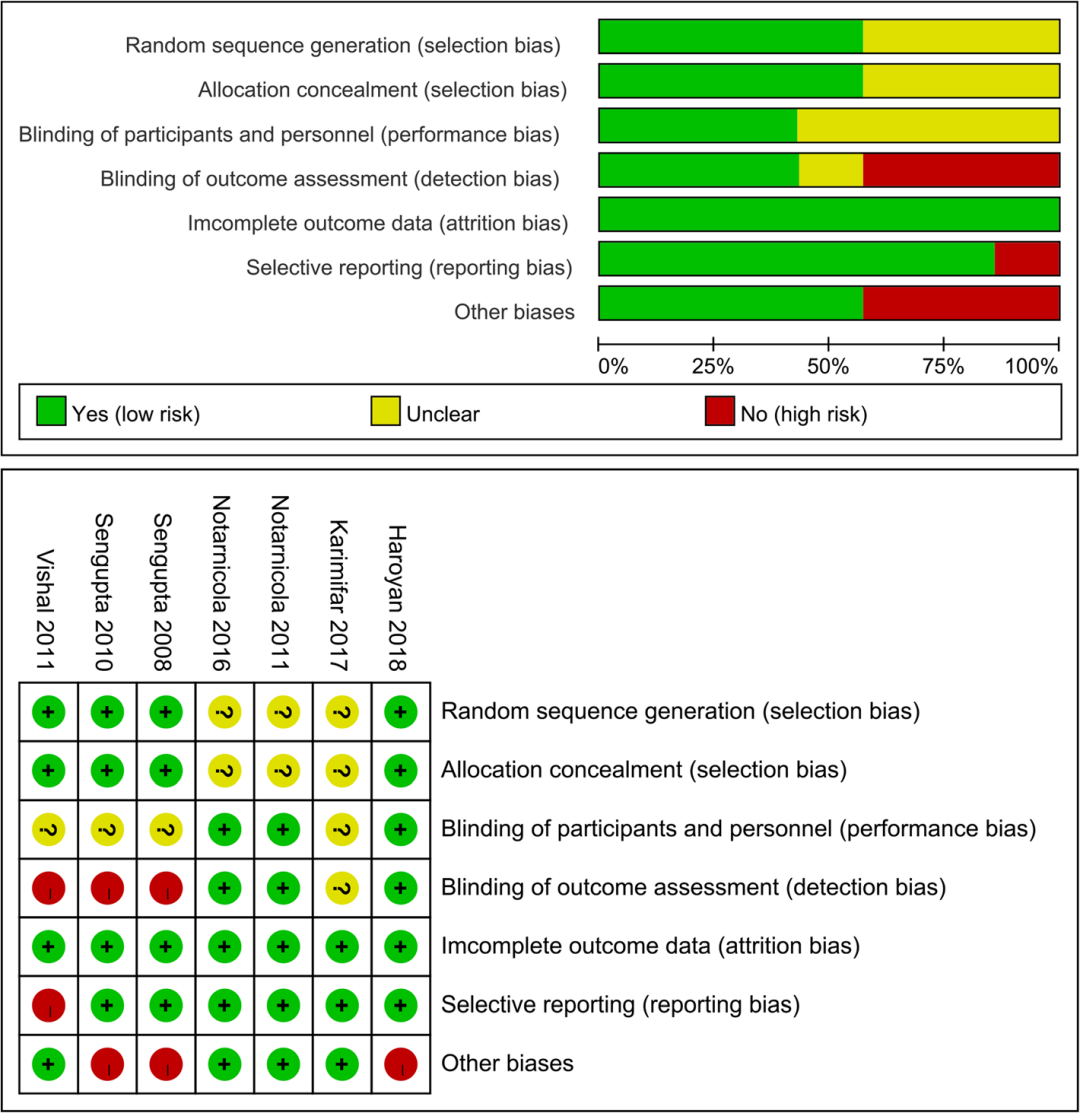
The risk of bias

The summary and graph of risk of bias ware shown in Fig. 2.

#### Sequence generation

Among the 7 included RCTs, three studies [35–37] adopted unclear randomization procedures, we therefore rated it as having an unclear risk of bias. The other RCTs described their randomization procedures: Sengupta 2010 [24], Sengupta 2008 [26] and Vishal 2011 [33] utilized the computer-generated randomization scheme, while Haroyan 2018 [34] used the treatment randomization code to draw randomization. Thus, these RCTs were thought to have low risks of bias.

#### Allocation concealment

Sengupta 2010 [24], Sengupta 2008 [26], Vishal 2011 [33] and Haroyan 2018 [34] described that the appearance, smell and color of drugs preparations were similar and organoleptically indistinguishable, which is an acceptable method of allocation concealment. Hence, they were rated as having low risks of bias. Karimifar 2017 [35], Notarnicola 2016 [36] and Notarnicola 2011 [37] did not describe an acceptable method of allocation concealment; therefore, they were rated as having an unclear risk of bias.

#### Blinding

For blinding of participants and personnel, although all RCTs claim to use blinding, only Haroyan 2018 [34], Notarnicola 2016 [36] and Notarnicola 2011 [37] described the implementation process of blinding. Thus, we gave a low risk of bias for them. The other RCTs were rated as having an unclear risk of bias for they did not describe the blind implementation process.

For blinding of outcome assessment, the statisticians in Sengupta 2010 [24], Sengupta 2008 [26] and Vishal 2011 [33] wasn’t blinded, hence, they were rated as having a high risk of bias. Haroyan 2018 [34], Notarnicola 2016 [36] and Notarnicola 2011 [37] described the implementation process of blinding, hence, we gave a low risk of bias for them. Karimifar 2017 [35] were rated as having an unclear risk of bias for they did not describe the blind implementation process.

#### Incomplete outcome data

The missing outcome data of all RCTs balanced in numbers across intervention groups with similar reasons for missing data across groups. We gave them low risks of bias.

#### Selective reporting

One RCT (Vishal 2011 [33]) failed to provide all outcomes mentioned in its protocols, thus we thought its risk of bias was high. The other RCTs provided their protocols, and all of the study’s pre-specified outcomes that are of interest in the review had been reported in the pre-specified way; their risks of bias were low.

#### Other potential bias

In the RCTs of Sengupta 2010 [24], Sengupta 2008 [26] and Vishal 2011 [33], their protocols noted three primary outcome parameters, which means that when p = 0.05/3 = 0.017, the difference is significant. Strictly speaking, the statistics of this study were not carried out correctly. Hence, their risks of bias were high. Similarly, in Haroyan 2018 [34]‘s RCT, the difference is significant when p = 0.05 / 2 = 0.025. Its risk of bias was also high. Other sources of bias were not observed in other RCTs; therefore, the risks of other bias of them were low.

### Primary outcomes

#### Visual analogue score

**Fig. 3:**
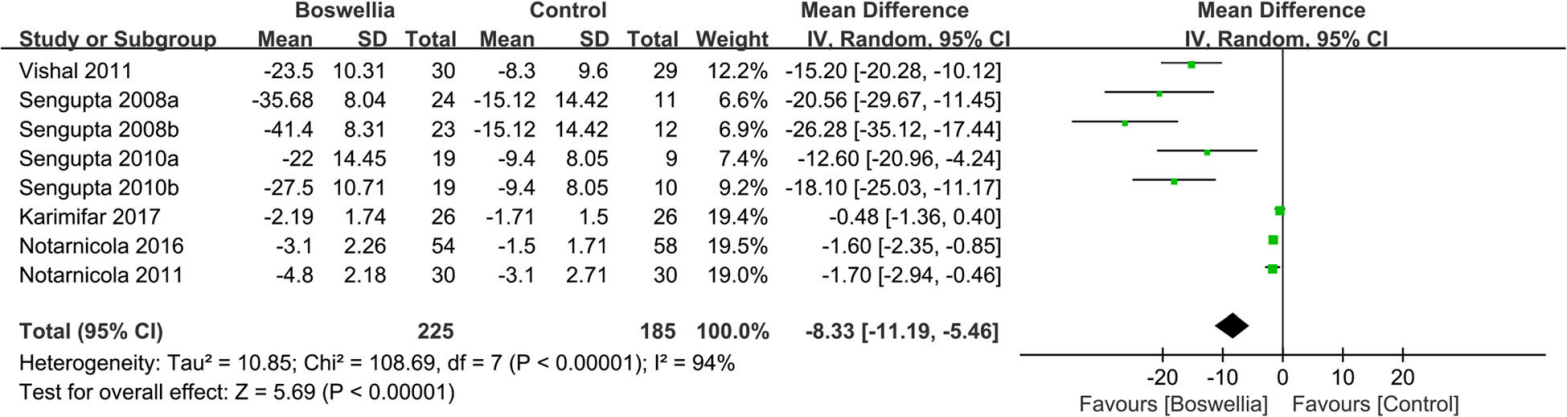
The changes of VAS

Six RCTs [24, 26, 33, 35–37] reported the changes of the visual analogue score (VAS) at the end of treatment. Due to the high heterogeneity (Tau^2^ = 10.85, I^2^ = 94%, *P*<0.00001), we used random effect model. In this index, it can be found that in improving VAS, Boswellia is better (WMD -8.33; 95% CI -11.19, − 5.46; *P*<0.00001). (Fig. 3).

#### WOMAC

**Fig. 4:**
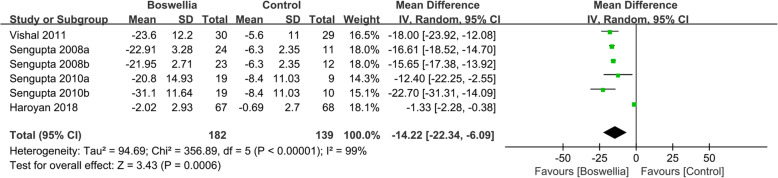
The changes of WOMAC pain

Four RCTs [24, 26-, 34–35] reported the changes of WOMAC pain. Due to the high heterogeneity (Tau^2^ = 94.69, I^2^ = 99%, *P*<0.00001), we used random effect model. According to the result, compared with the control group, Boswellia is better in improving WOMAC pain (WMD -14.22; 95% CI -22.34, − 6.09; P = 0. 0006). (Fig. 4).

**Fig. 5:**
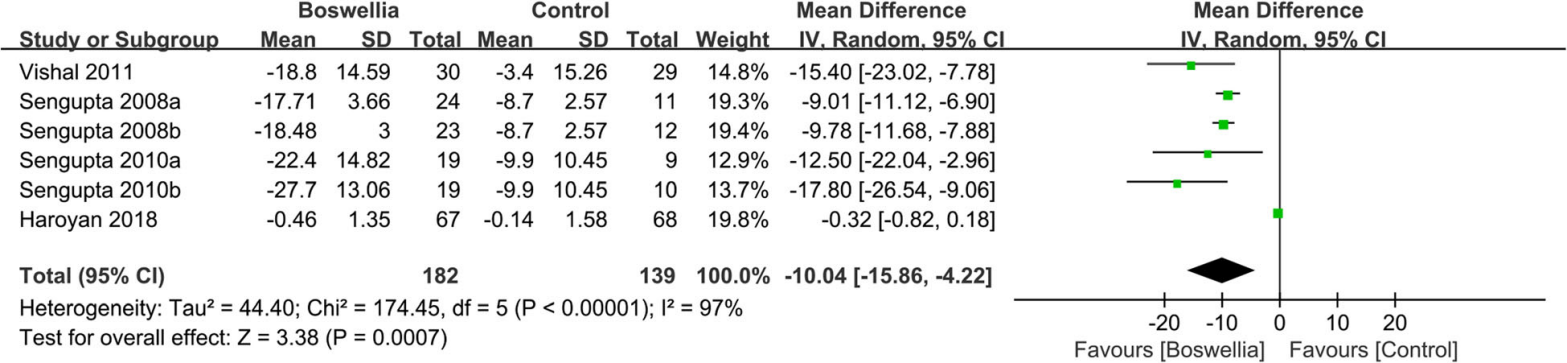
The changes of WOMAC stiffness

Four RCTs [24, 26, 33, 34] reported the changes of WOMAC stiffness. Due to the high heterogeneity (Tau^2^ = 44.40, I^2^ = 97%, *P*<0.00001), we used random effect model. According to the result, compared with the control group, Boswellia is better in improving WOMAC stiffness (WMD -10.04; 95% CI -15.86, − 4.22; P = 0. 0007). (Fig. 5).

**Fig. 6:**
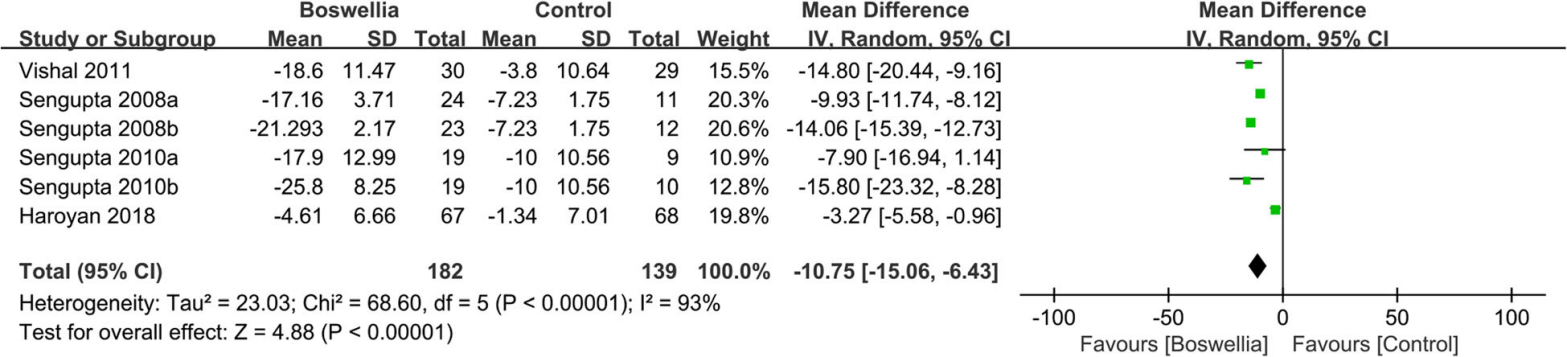
The changes of WOMAC function

Four RCTs [24, 26, 33, 34] reported the changes of WOMAC function. Due to the high heterogeneity (Tau^2^ = 23.03, I^2^ = 93%, *P*<0.00001), we used random effect model. According to the result, compared with the control group, Boswellia is better in improving WOMAC function (WMD -10.75; 95% CI -15.06, − 6.43; *P*<0.00001). (Fig. 6).

#### Lequesne index

**Fig. 7:**
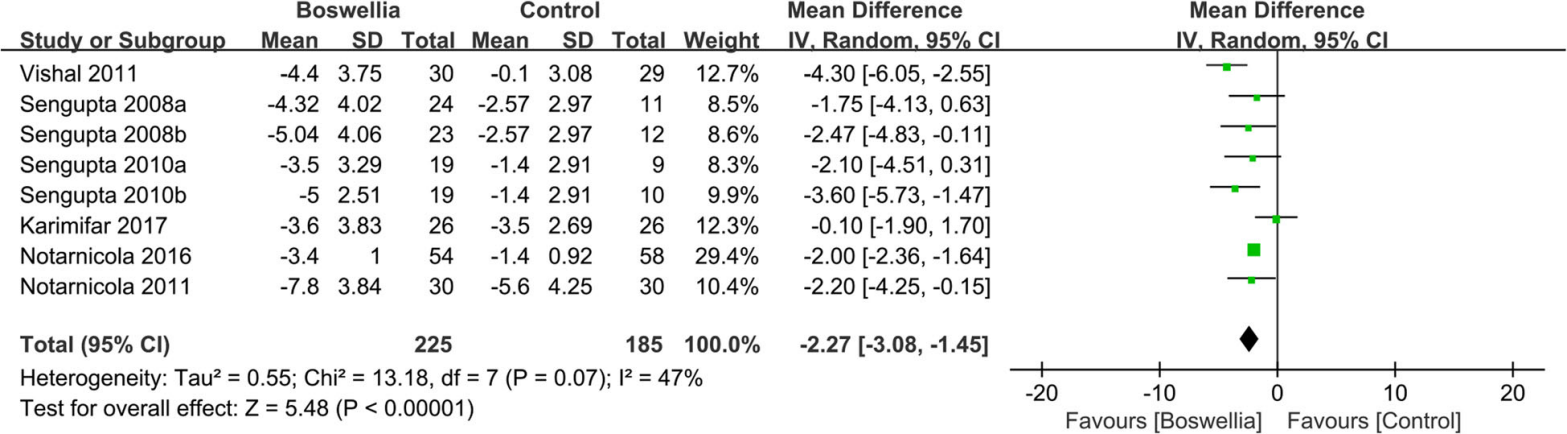
The changes of Lequesne Index

Six RCTs [24, 26, 33, 35–37] reported the changes of lequesne index. Due to the high heterogeneity (Tau^2^ = 0.55, I^2^ = 47%, P = 0.07), we used random effect model. According to the result, compared with the control group, Boswellia is better in improving Lequesne Index (WMD -2.27; 95% CI -3.08, − 1.45; *P*<0.00001). (Fig. 7).

### Secondary outcomes

#### Pain

Several RCTs reported Pain index (VAS and/or WOMAC pain) at week 4, 8, 12. The details of them were shown in Table 4 and Figure S1 ~ S6 (see Supplementary Materials).

**Table 4:** the pain index at week 4, 8, 12

Secondary outcomes	Overall effect						Heterogeneity test			Figure	Reference
	MD	95%CI	P	Tau^2^	I^2^ (%)	P	Statistical method	Studies (N)	Sample size (N)		
VAS at 4 weeks	−6.38	−10.20, −2.57	0.001	18.94	95%	<0.00001	Random inverse variance	4	238	Fig S1	[24, 26, 33, 35]
VAS at 8 weeks	−5.44	−8.8, −2.09	0.001	14.11	95	<0.00001	Random inverse variance	4	299	Fig S2	[24, 26, 36, 37]
VAS at 12 weeks	−19.37	−23.90, − 14.85	<0.00001	–	43	0.15	Fixed inverse variance	2	127	Fig S3	[24, 26]
WOMAC pain at 4 weeks	−6.91	− 11.08, −2.74	0.001	15.85	85	<0.0001	Random inverse variance	3	186	Fig S4	[24, 26, 33]
WOMAC pain at 8 weeks	−11.05	−14.94, −7.17	<0.00001	10.57	79	0.003	Random inverse variance	2	127	Fig S5	[24, 26]
WOMAC pain at 12 weeks	−12.37	−19.47, −5.26	0.0006	58.02	97	<0.00001	Random inverse variance	3	262	Fig S6	[24, 26, 34]

#### Stiffness

Several RCTs reported Stiffness index (WOMAC stiffness) at week 4, 8, 12. The details of them were shown in Table 5 and Figure S7 ~ S9 (see Supplementary Materials).

**Table 5:** the stiffness index at week 4, 8, 12

Secondary outcomes	Overall effect						Heterogeneity test			Figure	Reference
	MD	95%CI	P	Tau^2^	I^2^ (%)	P	Statistical method	Studies (N)	Sample size (N)		
WOMAC stiffness at 4 weeks	− 5.10	− 8.55, − 1.64	0.004	8.23	64	0.03	Random inverse variance	3	186	Fig S7	[24, 26, 33]
WOMAC stiffness at 8 weeks	− 8.02	− 11.75, − 4.29	<0.0001	7.64	60	0.06	Random inverse variance	2	127	Fig S8	[24, 26]
WOMAC stiffness at 12 weeks	− 10.97	− 19.30, − 2.63	0.010	82.6	99	<0.00001	Random inverse variance	3	262	Fig S9	[24, 26, 34]

#### Function

Several RCTs reported Function index (WOMAC function) at week 4, 8, 12. The details of them were shown in Table 6 and Figure S10 ~ S12 (see Supplementary Materials).

**Table 6:** the function index at week 4, 8, 12

Secondary outcomes	Overall effect						Heterogeneity test			Figure	Reference
	MD	95%CI	P	Tau^2^	I^2^ (%)	P	Statistical method	Studies (N)	Sample size (N)		
WOMAC pain at 4 weeks	− 7.18	− 10.56, − 3.80	<0.0001	9.23	81	0.0003	Random inverse variance	3	186	Fig S10	[24, 26, 33]
WOMAC pain at 8 weeks	− 8.95	−13.56, − 4.35	0.0001	14.91	84	0.0004	Random inverse variance	2	127	Fig S11	[24, 26]
WOMAC pain at 12 weeks	− 12.79	−18.41, − 7.43	<0.00001	22.56	94	<0.00001	Random inverse variance	3	262	Fig S12	[24, 26]

### Adverse events

**Fig. 8:**
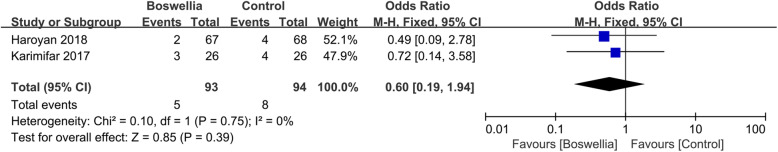
Adverse events

Five studies [24, 33–35, 37] reported AEs. Three of them were excluded because they reported no events in both arms. According to the results, there is also not strong evidence that which one is safer because there was no statistical difference (RR 0.63; 95% CI 0.22, 1.83; P = 0.39) (Fig. 8).

## Discussions

This systematic review and meta-analysis including 7 RCTs analyzes the effectiveness and safety of Boswellia and its extract for OA. Compared with the control group, Boswellia and its extract may relieve the pain (VAS and WOMAC pain) and stiffness (WOMAC stiffness), and improve the joint’s function (WOMAC function and lequesne index). In particular, since the doses of Boswellia and its extract reported in RCTs used for secondary outcomes analysis are 100–250 mg, based on the current evidence, pain, stiffness and joints’ function start to improve after 4 weeks of continuous Boswellia and its extract (at least 100–250 mg) intervention. While this finding seems promising, it should be interpreted with caution mainly due to the unclear risk of bias for selection bias (random sequence generation, allocation concealment) and attrition bias (incomplete outcome data), the high risk of bias for reporting bias (selective reporting), and the small number of participants.

Five studies [24, 33–35, 37] reported AEs. Three of them were excluded because they reported no events in both arms. According to the results, there is also not strong evidence that which one is safer because there was no statistical difference. However, safety studies conducted according to OECD guidelines and extensive acute and dose-dependent subchronic safety experiments on rats demonstrate that Boswellia and its extract does not exhibit toxic manifestations [23, 25], which means Boswellia and its extract might be a safety treatment option. But the absence of information on AEs does not mean that the intervention is safe [43]. Thus, although based on current evidences, we consider that Boswellia and its extract is a relatively safe treatment, we cannot assure it. Future clinical trials are required to report AEs with more explanations [44]. Thus, although based on current evidences, we consider that Boswellia and its extract is a relatively safe treatment, we cannot assure it. Future clinical trials are required to report AEs with more explanations [45].

Although there were several meta-analyses that have been widely analyzed the effect of herb or dietary supplements on OA [28, 29], as far as we know, this systematic review and meta-analysis is the first one that focused on evaluating the efficacy and safety of Boswellia and its extract for patients with OA. Compared with similar previous works [28, 29], we included more RCTs (including [33–36]) about Boswellia and its extract; thus, our conclusions were more accurate and reliable. In particular, compared with the work of Cameron et al. [29], we not only verified some outcomes (such as WOMAC) but also provide the recommended duration and dosage of Boswellia and its extract and so on. However, there are still some deficiencies in this study. For example, the risk of bias of some RCTs are high; the number of RCTs and cases included in this study is insufficient; the heterogeneity of some outcomes is high; these all affect the accuracy of the conclusions. The heterogeneity may be related to the discrepancies in the pharmacological effects of different Boswellia preparations. This may result from the different standardization of Boswellia and its extract manufacturing process, dosage, duration of treatment, units of laboratory tests and races of the selected patients or other places. Meanwhile, the recommended duration and dosage of treatment should also be interpreted with caution because the pain, stiffness and function index between week 1 and week 4 and the dosage of Boswellia and its extract outside 100–250 mg were not reported. Additionaly, since the quality of the RCTs is generally medium to low, all results should be interpreted more cautiously. Last but not least, further rigorously designed studies with high quality are needed to confirm the effectiveness and safety of Boswellia and its extract preparations for patients with OA.

## Conclusion

This systematic review and meta-analysis showed that Boswellia and its extract may be a novel drug for patient with OA. Based on current evidence, the recommended duration and dosage of treatment with Boswellia and its extract is at least 100-250 mg 4 weeks. However, current RCTs have limitations, including the missing information of pain, stiffness and function index between week 1 and week 4 and small sample sizes. Meanwhile, since the quality of the RCTs is generally medium to low, we should formulate the conclusion more cautiously. More double-blind, large sample size RCTs of Boswellia and its extract for OA that reported pain, stiffness and function index before 4 weeks and the dosage of Boswellia and its extract outside 100–250 mg are needed in the future to confirm or modify the result of this work.

## Supplementary information

**Additional file 1.**

**Additional file 2.**

**Additional file 3.**

**Additional file 4.**

**Additional file 5.**

**Additional file 6.**

**Additional file 7.**

**Additional file 8.**

**Additional file 9.**

**Additional file 10.**

**Additional file 11.**

**Additional file 12.**

## Data Availability

All data generated or analyzed during this study are included in this published article and its supplementary information files.

## Abbreviations

OA: Osteoarthritis
NSAIDs: Non-steroidal anti-inflammatory drugs
MMP: Matrix metalloproteinase
RCT: Randomized controlled trials
AE: Adverse events
